# Gender-Specific Long-Term Results After Elective Open Abdominal Aortic Aneurysm Repair Depending on the Site of Distal Anastomosis

**DOI:** 10.1177/15385744241276702

**Published:** 2024-08-28

**Authors:** Sonny Gennaro Annunziata, Jasmin Epple, Thomas Schmitz-Rixen, Dittmar Böckler, Reinhart T. Grundmann

**Affiliations:** 1 Department of Vascular and Endovascular Surgery, University Hospital, Frankfurt am Main, Germany; 2 Department of Vascular and Endovascular Surgery, 9173 Goethe University Frankfurt, Frankfurt am Main, Germany; 3 Department of Vascular and Endovascular Surgery, University Hospital Heidelberg Germany; 4 Department of Vascular Medicine, University Heart and Vascular Center (UHC), University Medical Center Hamburg-Eppendorf, Hamburg, Germany

**Keywords:** open aneurysm repair, gender, tube graft, biiliac bifurcated graft, bifemoral bifurcated graft, survival

## Abstract

**Objective:**

Analysis of gender-specific differences in short- and long-term outcome after elective open abdominal aortic aneurysm repair (OAR) regarding the distal anastomosis.

**Methods:**

In this retrospective cohort study, data from 4853 patients of a German health insurance company undergoing OAR for infrarenal abdominal aortic aneurysms (AAAs) between 2010 and 2016 were analysed. The patients were followed through 2018.

**Results:**

A total of 4050 (83.5%) men and 803 (16.6%) women underwent OAR. Women were older than men (72.9 ± 8.7 vs 69.8 ± 8.5 years; *P* < .001). A tube graft was used in 2644 (54.5%) patients, an aorto-biiliac bifurcated graft in 1657 (34.1%) and an aorto-bifemoral bifurcated graft in 552 (11.4%). Perioperative mortality was not significantly different between men (5.7%) and women (6.5%) in the total patient population (*P* = .411). This was true for aorto-aortic tube grafting (*P* = .361), aorto-biiliac reconstructions (*P* = 1.000) and aorto-bifemoral reconstructions (*P* = .345). Kaplan-Meier estimated long-term survival of men after 9 years was better than that of women (55.0% vs 43.8%; *P* = .006). However, separated by the site of the distal anastomosis, this was only true for aorto-aortic reconstructions (survival men vs women 56.0% vs 42.1%; *P* = .005), not for aorto-biiliac and aorto-bifemoral reconstructions. In the multivariate Cox regression analysis, age over 80 years, heart failure, aorto-bifemoral reconstruction, chronic kidney disease stage 3-5, chronic obstructive pulmonary disease, peripheral artery disease, arterial hypertension, but not gender (*P* = .531), had a negative impact on long-term survival.

**Conclusion:**

If possible, an aorto-aortic tube graft should be preferred to aorto-biiliac and aorto-bifemoral reconstructions in OAR. Patients selected for aorto-bifemoral artery reconstruction exhibit higher perioperative morbidity and mortality as well as worse long-term survival compared to patients selected for an intra-abdominal reconstruction. In the multivariate regression analysis, gender was not an independent risk factor for either short- or long-term outcomes.

## Introduction

This paper considers gender-specific differences in long-term outcomes after open abdominal aortic aneurysm repair (OAR) with respect to aortic reconstruction. The advantages of tube vs bifurcation graft replacement of abdominal aortic aneurysms are well known (shorter procedure time and less blood loss), so that bifurcated grafts can be reserved for patients with concomitant iliac aneurysms or aorto-iliofemoral occlusive disease (Friedman et al^1^ 1990; Calcagno^2^ 1991; Ballotta^3^ 2008; Hassen-Khodja^4^ 2006). A large study on the long-term outcome after open surgical repair of abdominal aortic aneurysms depending on the site of the distal anastomosis has only recently been published by King et al^5^ (2023). It could be demonstrated that more proximal sites of distal anastomosis should be selected in OAR when technically feasible. Aorto-aortic tube grafting was associated with fewer perioperative complications when compared with bifurcated reconstructions. However, no gender specific differences in outcome were analyzed. Gender disparities in outcome were reported in patients undergoing aorto-bifemoral bypass (ABFB) or aortic thromboendarterectomy (ATEA) for aortoiliac disease (Lombardi et al^6^ 2023). Although there was no sex-based mortality difference at 30 days, there was a statistically significant increase in mortality in females after open aortoiliac intervention at 1 year. In the present study, therefore we examined whether short- and long-term outcomes of men and women differ after OAR depending on the choice of distal anastomosis (aorto-aortic, aorto-biiliac, aorto-bifemoral) using data from of the largest German health insurance company.

## Methods

The data analyzed in this retrospective study were provided anonymously by the Scientific Institute of the AOK (WidO). The study involved 4853 patients with intact abdominal aortic aneurysm (iAAA; ICD code: I71.4) who underwent open aneurysm repair throughout Germany between January 1, 2010 and December 31, 2016. The data were analyzed separately by patient gender and type of graft used: Group A: Abdominal aorta, infrarenal: tube graft (OPS: 5-384.7(1-2)), Group B: Abdominal aorta, infrarenal: aorto-biiliac bifurcation graft (OPS: 5-384.7(3-4)) and Group C: Abdominal aorta, infrarenal: aorto-bifemoral bifurcation graft (OPS: 5-384.7(5-6)). 38 patients were excluded because they could not be assigned to a specific type of prosthesis (OPS: 5-384.7x). The three groups were analyzed with regard to gender, comorbidities, perioperative complications and hospital mortality, long-term survival and complication rates at follow-up. The follow-up period ended on December 31, 2018. On average, the patients were followed up for 79 months (minimum follow-up: 0 months, maximum follow-up 107 months) postoperatively.

### Statistical Analysis

The patient information from the AOK datasets was analyzed in a database created using SPSS (IBM Deutschland GmbH, Ehningen, Germany). To test the groups for differences, the chi-square test was used for nominal and categorical variables. The Mann-Whitney-U-Test was used for metric variables. The Kaplan-Meier analysis and the log-rank test were used to compare survival times. To analyze the influence of patient characteristics such as age and gender, comorbidities and choice of the distal anastomosis on short and long-term survival, a univariate Cox regression was performed. The parameters for which a significant influence was found were then combined in a multivariate Cox regression. The significance level was defined as *P* < .05.

## Results

### Patients

The study population consisted of 4050 (83.5%) men and 803 (16.6%) women who underwent OAR. Women were significantly older than men (72.9 ± 8.7 vs 69.8 ± 8.5 years; *P* < .001). A tube graft was used in 2644 (54.5%) patients (group A), an aorto-biiliac bifurcation graft in 1657 (34.1%) (group B) and an aorto-bifemoral bifurcation graft in 552 (11.4%) (group C).

The tube graft was used significantly more frequently in women than in men (64.3% vs 52.6%; *P* < .001). In contrast, the biiliac bifurcation prosthesis was used more frequently in men (men: 36.3%, women: 23.5%, *P* < .001). The bifemoral bifurcation prosthesis was used equally frequently in both genders (men: 11.2%, women: 12.2%, *P* = .429). In comorbidities, men differed from women only in a higher history of myocardial infarction (9.4% vs 7.1%; *P* = .043). The prevalence of all other comorbidities was similar for both sexes (Table 1).

**Table 1.** Patient Characteristics and Comorbidities in Men and Women.

	Men (n = 4050)	Women (n = 803)	*P*-value
Tube graft, n (%) (n = 2644)	2128 (52.6%)	516 (64.3%)	<.001
Aorto-biiliac graft, n (%) (n = 1657)	1468 (36.3%)	189 (23.5%)	<.001
Aorto-bifemoral graft, n (%) (n = 552)	454 (11.2%)	98 (12.2%)	.429
Age, mean SD, median (min-max)	69.8 8.5 71.0 (33-96)	72.9 8.7 74.0 (16-91)	<.001
History of myocardial infarction, n (%)	379 (9.4%)	57 (7.1%)	.043
History of stroke, n (%)	162 (4.0%)	22 (2.7%)	.105
History of TIA, n (%)	79 (2.0%)	16 (2.0%)	.890
Arterial hypertension, n (%)	1722 (42.5%)	354 (44.1%)	.413
Diabetes mellitus type 2, n (%)	474 (11.7%)	89 (11.1%)	.673
COPD, n (%)	410 (10.1%)	99 (12.3%)	.067
Chronic kidney disease (stage 3-5), n (%)	296 (7.3%)	68 (8.5%)	.271
Heart failure (NYHA 3-4), n (%)	214 (5.3%)	45 (5.6%)	.731
PAD (stage 3 and 4), n (%)	158 (3.9%)	24 (3.0%)	.263

SD, standard deviation; min-max: minimum-maximum, TIA, transient ischemic attack; COPD, chronic obstructive pulmonary disease, chronic kidney disease: stage 3-5: glomerular filtration rate under 60 ml/min/1.73 m^2^; NYHA, New York Heart Association; PAD, peripheral artery disease.

The patient cohort with a tube graft was significantly older (71.1 ± 8.5 years) than patients with biiliac (69.1 ± 9.0) and bifemoral (69.8 ± 8.0) reconstructions (*P* < .001). The percentage of patients with Fontaine stage III and IV peripheral artery disease (PAD) was significantly higher with bifemoral reconstructions than with biiliac and aorto-aortic reconstructions (7.6% vs 3.8% vs 2.9%; *P* < .001). Iliac aneurysms were found in 0.5% of patients with aorto-aortic reconstructions, but in 5.9% of patients with biiliac reconstructions (*P* < .001). Patients with bifemoral reconstructions had significantly fewer iliac aneurysms (2.7%) than patients with biiliac reconstructions (*P* = .003) (Table 2).

**Table 2.** Patient Characteristics and Comorbidities in Association With the Distal Anastomoses.

	A Tube graft (n = 2644)	B Biiliac graft (n = 1657)	C Bifemoral graft (n = 552)	*P*-value
Age. Mean SD. median (min-max)	71.1 8.5 72 (38-96)	69.1 9.0 70 (16-95)	69.8 8.0 71 (47-93)	A vs B: *P* < .001 A vs C: *P* < .001 B vs C: *P* = .077
History of myocardial infarction. n (%)	232 (8.8%)	147 (8.9%)	57 (10.3%)	A vs B: *P* = .912 A vs C: *P* = .253 B vs C: *P* = .309
History of stroke. n (%)	101 (3.8%)	50 (3.0%)	33 (6.0%)	A vs B: *P* = .174 A vs C: *P* = .026 B vs C: *P* = .003
History of TIA. n (%)	48 (1.8%)	39 (2.4%)	8 (1.4%)	A vs B: *P* = .223 A vs C: *P* = .721 B vs C: *P* = .236
Arterial hypertension. n (%)	1109 (41.9%)	713 (43.0%)	254 (46.0%)	A vs B: *P* = .486 A vs C: *P* = .080 B vs C: *P* = .235
Diabetes mellitus type 2. n (%)	310 (11.7%)	183 (11.0%)	70 (12.7%)	A vs B: *P* = .523 A vs C: *P* = .516 B vs C: *P* = .316
COPD. n (%)	298 (11.3%)	146 (8.8%)	65 (11.8%)	A vs B: *P* = .010 A vs C: *P* = .713 B vs C: *P* = .045
Chronic kidney disease (stage 3-5). n (%)	193 (7.3%)	124 (7.5%)	47 (8.5%)	A vs B: *P* = .857 A vs C: *P* = .329 B vs C: *P* = .462
Heart failure (NYHA 3-4). n (%)	218 (8.2%)	156 (9.4%)	56 (10.1%)	A vs B: *P* = .201 A vs C: *P* = .155 B vs C: *P* = .617
PAD (stage 3 and 4). n (%)	77 (2.9%)	63 (3.8%)	42 (7.6%)	A vs B: *P* = .113 A vs C: *P* < .001 B vs C: *P* < .001
*A. iliaca aneurysm*. n (%)	12 (0.5%)	97 (5.9%)	15 (2.7%)	A vs B: *P* < .001 A vs C: *P* < .001 B vs C: *P* = .003

*A*: OAR with tube grafts (OPS: 5-384.7(1-2)).

*B*: OAR with aorto-biiliac grafts (OPS: 5-384.7(3-4)).

*C*: OAR with aorto-bifemoral grafts al (OPS: 5-384.7(5-6))

SD, standard deviation; min-max, minimum-maximum; TIA, transient ischemic attack; COPD, chronic obstructive pulmonary disease; chronic kidney disease: stage 3-5: glomerular filtration rate under 60 ml/min/1.73 m^2^, NYHA, New York Heart Association; PAD, peripheral artery disease.

### Perioperative Outcome

Perioperative mortality was not significantly different between men (5.7%) and women (6.5%) in the overall patient population (*P* = .411). This was the case for aorto-aortic tube grafts (4.7% vs 5.6%; *P* = .361), aorto-biiliac reconstructions (6.3% vs 5.8%; *P* = 1.000) and aorto-bifemoral reconstructions (9.0% vs 12.2%; *P* = .345). In over 80-year-olds, hospital mortality was 14.2% in men and 15.7% in women (*P* = .625). The postoperative length of stay was significantly longer in women (21.5 ± 15.5 days) compared to men (20.0 ± 15.6 days; *P* < .001) and blood transfusions were given more often in women (72.7% vs 58.3%; *P* < .001). Complex intensive care treatment was required in 62.0% of women and 59.3% of men (*P* = .156). Major limb amputations were performed in 0.5% of men and 0.5% of women (*P* = 1.000). Wound infections were observed in 8.3% of men and 5.4% of women (*P* = .003). Blood transfusions were given in 71.1% of patients with wound infections but only in 59.8% of patients without wound infection (*P* < .001).

Patients with aorto-bifemoral reconstruction had a significantly higher hospital mortality (9.6%) than patients with tube grafts (4.8%; *P* < .001) and biiliac reconstructions (6.2%, *P* = .009). Patients with aorto-bifemoral reconstructions had a significantly longer hospital stay, higher rates of blood transfusions, acute renal failure, pneumonia, wound infection, embolism and thrombosis, and major limb amputation compared to the other reconstructions.

Compared to biiliac reconstructions, patients with aorto-aortic reconstructions had significantly lower in-hospital mortality in men (4.7% vs 6.3%; *P* = .041), but not in women (5.6% vs 5.8%; *P* = 1.000). In aorto-aortic reconstructions, the hospital stay was significantly shorter, and the blood transfusion rate was lower as compared to biiliac reconstructions. Wound infections were observed in 6.3% with aorto-aortic reconstructions, but in 8.8% with biiliac reconstructions (*P* = .003), embolism and thrombosis of the lower extremity in 3.1% vs 6.1% (*P* < .001). Complex intensive care treatment was required less frequently for aorto-aortic reconstruction than for biiliac reconstruction (57.5% vs 61.5%; *P* = .010). Further information on the perioperative outcome depending on the site of the distal anastomosis can be found in Table 3. In the multivariate binary logistic Cox regression analysis, age over 80 years, history of TIA, heart failure (NYHA 3-4), bifemoral reconstruction and CKD stage 3-5 had a significant negative impact on perioperative mortality (Table 4). Gender had no significant influence in the univariate regression (OR: 1.139, CI: 0.835-1.555, *P* = .410). Patients with a tube graft had a significantly better perioperative survival compared to the patients with a bifurcated graft (OR: 0.616, CI: 0.480-0.791, *P* < .001).

**Table 3.** Perioperative Mortality and Complications in Association With the Distal Anastomoses.

	A Tube graft (n = 2644)	B Biiliac graft (n = 1657)	C Bifemoral graft (n = 552)	*P*-value
Perioperative mortality, n (%)	128/2644 (4.8%)	103/1657 (6.2%)	53/552 (9.6%)	A vs B: *P* = .060 A vs C: *P* < .001 B vs C: *P* = .009
Perioperative mortality men, n (%)	99/2128 (4.7%)	92/1468 (6.3%)	41/454 (9.0%)	A vs B: *P* = .041 A vs C: *P* < .001 B vs C: *P* = .045
Perioperative mortality women, n (%)	29/516 (5.6%)	11/189 (5.8%)	12/98 (12.2%)	A vs B: *P* = 1.000 A vs C: *P* = .025 B vs C: *P* = .068
LOS, mean SD, median (min-max)	19.11 15.1 14 (1-327)	20.63 15.0 15 (1-137)	24.81 18.7 19 (0-176)	A vs B: *P* < .001 A vs C: *P* < .001 B vs C: *P* < 0.001
Blood transfusion, n (%)	1507 (57.0%)	1051 (63.4%)	388 (70.3%)	A vs B: *P* < .001 A vs C: *P* < .001 B vs C: *P* = .003
Complex intensive care treatment, n (%)	1520 (57.5%)	1019 (61.5%)	360 (65.2%)	A vs B: *P* = .010 A vs C: *P* < .001 B vs C: *P* = .128
Wound infections, n (%)	167 (6.3%)	146 (8.8%)	68 (12.3%)	A vs B: *P* = .003 A vs C: *P* < .001 B vs C: *P* = .020
Acute myocardial infarction, n (%)	74 (2.8%)	34 (2.1%)	22 (4.0%)	A vs B: *P* = .134 A vs C: *P* = .169 B vs C: *P* = .018
Cerebral complications, n (%)	34 (1.3%)	27 (1.6%)	12 (2.2%)	A vs B: *P* = .357 A vs C: *P* = .117 B vs C: *P* = .455
Dialysis, n (%)	127 (4.8%)	99 (6.0%)	56 (10.1%)	A vs B: *P* = .106 A vs C: *P* < .001 B vs C: *P* = .001
Acute renal failure, n (%)	209 (7.9%)	160 (9.7%)	82 (14.9%)	A vs B: *P* = .050 A vs C: *P* < .001 B vs C: *P* < .001
Pneumonia, n (%)	279 (10.6%)	144 (8.7%)	85 (15.4%)	A vs B: *P* = .051 A vs C: *P* = .002 B vs C: *P* < .001
Embolism and thrombosis of the lower extremities, n (%)	81 (3.1%)	101 (6.1%)	52 (9.4%)	A vs B: *P* < .001 A vs C: *P* < .001 B vs C: *P* = .012
Mesenteric ischemia, n (%)	59 (2.2%)	52 (3.1%)	19 (3.4%)	A vs B: *P* = .075 A vs C: *P* = .096 B vs C: *P* = .780
Amputation of the lower limb, n (%)	8 (0.3%)	8 (0.5%)	8 (1.4%)	A vs B: *P* = .441 A vs C: *P* = .003 B vs C: *P* = .036

*A*: OAR with tube grafts (OPS: 5-384.7(1-2)).

*B*: OAR with aorto-biiliac grafts (OPS: 5-384.7(3-4)).

*C*: OAR with aorto-bifemoral grafts al (OPS: 5-384.7(5-6)).

SD, standard deviation; min-max, minimum-maximum; LOS, length of stay; OAR, open abdominal aortic aneurysm repair.

**Table 4.** Results of Multivariate Logistic Binary Regression Analysis for Perioperative Mortality.

	Odds ratio	95% CI	*P*-value
Age over 80 years	3.664	2.806-4.785	<.001
History of TIA	2.879	1.587-5.223	<.001
Heart failure (NYHA 3-4)	2.122	1.399-3.220	<.001
Aorto-bifemoral vs tube and biiliac grafts	2.006	1.454-2.767	<.001
Chronic kidney disease (stage 3-5)	1.636	1.118-2.394	.011
Arterial hypertension	1.163	0.888-1.524	.273
Diabetes mellitus type 2	1.202	0.840-1.721	.314
Aneurysm iliac artery	0.876	0.413-1.855	.729

TIA, transient ischemic attack; NYHA, New York Heart Association; Chronic kidney disease stage 3-5: glomerular filtration rate under 60 ml/min/1.73 m^2^; PAD, peripheral artery disease; COPD, chronic obstructive pulmonary disease.

The following variables had no significant influence on perioperative mortality in the univariant regression analysis: women, history of myocardial infarction, history of stroke, PAD, COPD.

### Long-Term Survival and Reintervention Rate

**Figure 1.**
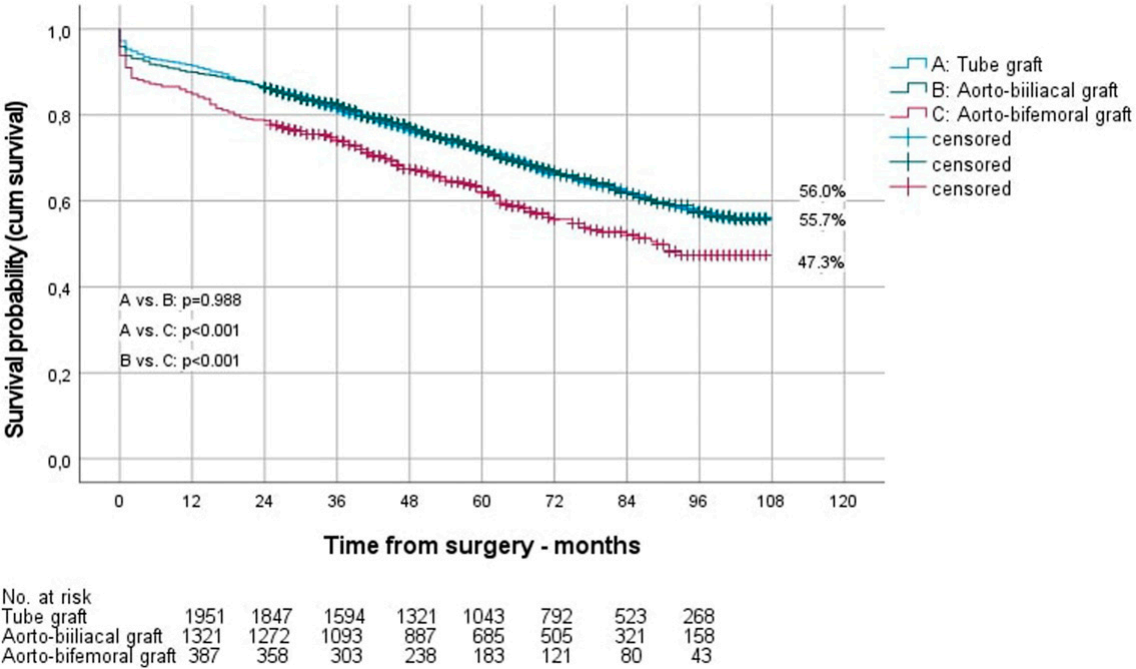
Long-term survival of men after OAR, depending on the choice of the distal anastomosis.

**Figure 2.**
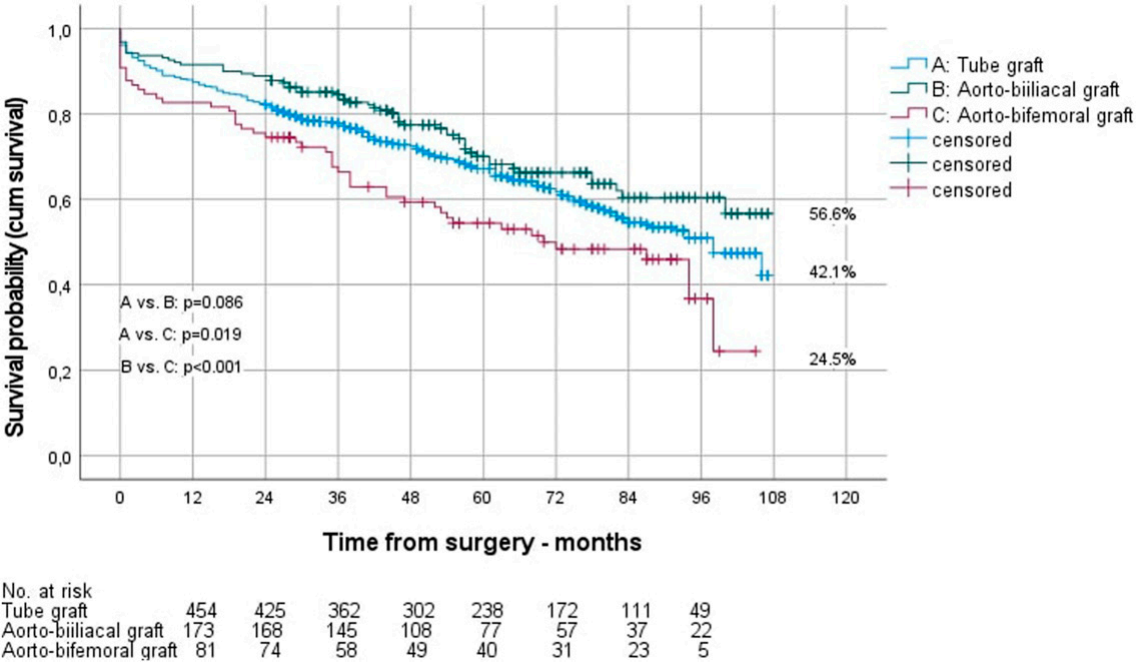
Long-term survival of women after OAR, depending on the choice of the distal anastomosis.

The long-term survival of men depending on the position of the distal anastomosis is shown in Figure 1, that of women in Figure 2.

Kaplan-Meier estimated long-term survival of men after 9 years was significantly better than that of women (55.0% vs 43.8%; *P* = .006). However, separated by the site of the distal anastomosis, this was only true for aorto-aortic reconstructions (survival men vs women 56.0% vs 42.1%; *P* = .005), not for aorto-biiliac (survival men vs women 55.7% vs 56.6%; *P* = .839) and aorto-bifemoral reconstructions (survival men vs women 47.3% vs 24.5%; *P* = .154). The reintervention rate during follow-up was 6.7% for men and 8.8% for women (*P* = .244). Readmissions per year were 1.5 ± 1.8 in men and 1.6 ± 2.2 in women, with a mean stay of 8.2 ± 7.9 vs 9.0 ± 7.4 days (*P* < .001).

Patients with bifemoral reconstructions had a significantly lower long-term survival (47.9%) than patients with tube grafts (53.3%; *P* < .001) and biiliac grafts (55.8%, *P* < .001). Patients with tube grafts underwent reinterventions significantly less frequently in the follow-up (5.8%) than those with biiliac reconstructions (8.1%; *P* = .003) and bifemoral reconstructions (10.1%; *P* < .001). Similar results were found for the reintervention rate in men, whereas in women there was no significant difference between aorto-aortic (7.0%) and aorto-biiliac anastomoses (10.7%, *P* = .072). Readmissions per year and the length of stay per readmission in the follow-up were significantly higher in patients who underwent aorto-bifemoral reconstruction. Further information on the long-term outcome depending on the site of the distal anastomoses are given in Table 5.

**Table 5.** Long-Term Survival and Reintervention Rate Over Nine-Year Follow-Up.

	A Tube graft (n = 2644)	B Biiliac graft (n = 1657)	C Bifemoral graft (n = 552)	*P*-value
Overall survival of all patients, n (%)	1741/2644 (53.3%)	1129/1657 (55.8%)	314/552 (47.9%)	A vs B: *P* = .368 A vs C: *P* < .001 B vs C: *P* < .001
Overall survival, men, n (%)	1426/2128 (56.0%)	998/1468 (55.7%)	266/454 (47.3%)	A vs B: *P* = .988 A vs C: *P* < .001 B vs C: *P* < .001
Overall survival, women, n (%)	315/516 (42.1%)	131/189 (56.6%)	48/98 (24.5%)	A vs B: *P* = .086 A vs C: *P* = .019 B vs C: *P* < .001
Reintervention rate, men, n (%)	65/82 (5.5%)	67/79 (7.8%)	25/33 (8.8%)	A vs B: *P* = .011 A vs C: *P* = .002 B vs C: *P* = .228
Reintervention rate, women, n (%)	17/82 (7.0%)	12/79 (10.7%)	8/33 (15.9%)	A vs B: *P* = .072 A vs C: *P* = .010 B vs C: *P* = .407
Readmissions per year, mean SD, median (min-max)	1.5 2.0 0.89 (0.11-24)	1.4 1.6 0.92 (0.11-18)	1.6 1.7 1.1 (0.11-12)	A vs B: *P* = .291 A vs C: *P* < .001 B vs C: *P* = .002
LOS per readmission, mean SD, median (min-max)	8.2 7.7 6.5 (0-134.5)	8.3 8.07 6.5 (0-102)	8.96 7.5 7.2 (0-57)	A vs B: *P* = .754 A vs C: *P* = .006 B vs C: *P* = .004

*A*: OAR with tube grafts (OPS: 5-384.7(1-2)).

*B*: OAR with aorto-biiliac grafts (OPS: 5-384.7(3-4)).

*C*: OAR with aorto-bifemoral grafts al (OPS: 5-384.7(5-6)).

SD, standard deviation; min-max, minimum-maximum; LOS, length of stay; OAR, open abdominal aortic aneurysm repair.

The percentages are Kaplan-Meier estimates.

In the multivariate regression analysis, age over 80 years, heart failure (NYHA 3-4), chronic kidney disease CKD 3-5, the use of an aorto-bifemoral graft, COPD, perioperative wound infections, PAD and arterial hypertension had a negative impact on long-term survival (Table 6).

**Table 6.** Multivariate Hazard Ratios (HR) for Long-Term Survival.

	HR	95% CI	*P*-value
Age over 80 years	2.513	2.235-2.826	<.001
Heart failure (NYHA 3-4)	1.586	1.304-1.928	<.001
Chronic kidney disease (stage 3-5)	1.461	1.236-1.726	<.001
Aorto-bifemoral vs tube and biiliac grafts	1.454	1.265-1.670	<.001
COPD	1.435	1.240-1.662	<.001
Perioperative wound infection	1.371	1.168-1.609	<.001
PAD (stage 3-4)	1.357	1.081-1.705	.009
Arterial hypertension	1.141	1.020-1.276	.021
Women (vs men)	1.053	0.929-1.194	.421
History of myocardial infarction	1.012	0.851-1.204	.889
History of stroke	1.200	0.932-1.546	.158
History of TIA	1.198	0.858-1.673	.289
Aneurysm iliac artery	1.075	0.808-1.430	.620
Diabetes mellitus type 2	1.033	0.888-1.202	.675

NYHA, New York Heart Association; Chronic kidney disease stage 3-5: glomerular filtration rate under 60 ml/min/1.73 m^2^; COPD, chronic obstructive pulmonary disease; PAD, peripheral artery disease; TIA, Transient ischemic attack.

## Discussion

In the present study, short- and long-term outcomes after elective OAR were analyzed with respect to aortic reconstructions and patient gender. Perioperative mortality was not significantly different for men (5.7%) and women (6.5%) in the overall patient cohort (*P* = .411). This was found likewise for aorto-aortic tube grafts, aorto-biiliac reconstructions and aorto-bifemoral reconstructions.

In contrast, Pouncey et al^7^ (2021) found in a systematic review with meta-analysis of 13 studies (including 12 374 women, 50 387 men) the sex-related 30-day mortality for OAR ranging from 3.64%-9.53% for women and 1.35%-6.81% for men. Cumulative analysis demonstrated statistically significantly higher odds of death in women. In a retrospective study from the Dutch Surgical Aneurysm Audit (DSAA) registry (2013-2018), a significantly higher hospital mortality rate after elective OAR was also observed in women as compared to men (6.9% vs 4.6%; *P* = .024) (Tedjawirja et al^8^ 2022). Desai et al^9^ (2016) reported sex-related trends in mortality after elective abdominal aortic aneurysm surgery between 2002 and 2013 at National Health Service hospitals in England. This study included 4795 women and 26 295 men with OAR. Women undergoing elective AAA repair had increased short- and long-term mortality and post-operative morbidity compared with men. The 30-day mortality (women vs men) was 6.65% vs 4.52% (*P* < .001) and 5 years mortality (excluding 30 days) was 10%–16% vs 6.66% (*P* < .001), resp. Finally, Cheng et al^10^ (2023) observed a significantly higher mortality rate in women compared to men in the first 48 hours after open AAA treatment in the Vascular Quality Initiative database.

In the current analysis, after nine years, Kaplan-Meier estimated long-term survival in the total population, was significantly (*P* = .006) better in men (55%) compared to women (43.8%) The reasons for this must remain open, as there was no difference between men (3.9%) and women (3.0%) with regard to the share of patients with PAD. However, women were significantly older than men. The risk-adjusted multivariate Cox regression analysis therefore found no significant differences in long-term survival between men and women (HR 1.053; CI 0.929-1.194; *P* = .421), sex was not an independent risk factor for long-term survival. Only in patients with tube grafts significantly better results were observed in men compared to women (overall survival after 9 years in men 56.0%, in women 42.1%; *P* = .005), where tube grafts were used relatively more frequently in women (64.3%) than in men (52.6%). In a retrospective cohort study (Ramkumar et al^11^ 2020) surgical AAA repair was performed in 27% (983 of 3629) of women compared with 18% (2328 of 12 757) of men (*P* < .001). In this study, the 10-year unadjusted survival rates were comparable in men and women after open surgical repair (36% in men vs 32% in women; log-rank *P* = .22). Risk-adjusted analysis showed that both men and women had a similar risk of death after open surgical repair (hazard ratio 0.94; 95%CI, 0.84-1.06). This contradicts data from the Swedish National Patient Registry (NPR) (Bulder et al^12^ 2020). This nationwide evaluation of survival after elective AAA repair between 2001 and 2015 showed alarming poor long-term prognosis, in particular for women, in comparison to the survival for the age- and sex-matched general population (relative survival). 4-year relative survival in three time periods was 0.78, 0.80, 0.70 in females vs 0.89, 0.89, 0.91, in males, respectively.

In the present analysis, patients with aorto-bifemoral reconstructions had a significantly lower long-term survival (47.9%) compared to patients with tube grafts (53.3%; *P* < .001) and biiliac grafts (55.8%, *P* < .001). Inversely, patients with tube grafts were significantly less likely to undergo reintervention over the long-term (5.8%) than those with biiliac (8.1%; *P* = .003) and bifemoral reconstructions (10.1%; *P* < .001). Patient preoperative risk factors do not explain these differences in results; patients with bifemoral prostheses were not older and did not show a higher rate of preoperative risk factors than patients in the other two groups. In their 2023 multicenter retrospective analysis, King et al presented data collected prospectively by the Vascular Quality Initiative. From 5683 patients with elective OAR, 40.4% underwent distal anastomosis to the aorta, 38.1% underwent distal anastomosis to the common iliac artery (CIA), 9.7% underwent distal anastomosis to the external iliac artery (EIA), and 11.8% underwent distal anastomosis to the common femoral artery (CFA). Distal anastomosis to the CFA was associated with increased odds of major in-hospital complications and reduced long-term survival. However, no significant differences in 30-day mortality across sites of distal anastomosis were observed. A distinction in the long-term outcomes of men and women was not performed by King et al.^5^ The conclusion was that in cases where anatomy and disease status permit, surgeons should feel comfortable prioritizing aorto-aortic tube grafting for its shorter length of procedure and relatively low risk of perioperative complications.

The presented results confirm this recommendation showing not only the long-term results (Figures 1 and 2) but also the perioperative results significantly worse in patients with aorto-bifemoral reconstructions (mortality: 9.6%) compared to aorto-aortic (mortality: 4.8%, *P* < .001) and aorto-biiliac (mortality: 6.2%, *P* = .009) reconstructions (Table 3).

Furthermore, the wound infection rate of 12.3% for bifemoral reconstructions was almost twice as high as for tube grafts at 6.3% (*P* < .001) and also significantly higher than that of biiliac grafts (8.8%, *P* = .003). York et al^13^ (2013) reported for surgical treatment of patients with aortoiliac occlusive disease groin wound complications in 0% for aorto-biiliac, but 15% for aorto-bifemoral reconstructions, however, the abdominal wound complications did not differ (11% vs 12%). Langenberg et al^14^ (2020) also found significantly higher wound infection rates with inguinal incisions in open aortoiliac surgery.

Restrictive it must be emphasized that it was not possible to examine to what extent the aorto-bifemoral reconstructions were due to the anatomical situation and to what extent a tube graft was preferred on principle to aorto-biiliac or aorto-bifemoral reconstructions when feasible. The choice of a bifurcated graft (aorto-biiliac or aorto-bifemoral) is a result of patient’s comorbidities and aneurysm anatomy. In this study, the percentage of patients with PAD stage III and IV (Fontaine) was significantly higher in aorto-bifemoral reconstructions than in patients with the other distal anastomoses (7.6% vs 3.8% vs 2.9%; *P* < .001). The percentage of patients with a history of stroke was significantly higher in patients with aorto-bifemoral grafts as well (tube graft: 3.8% vs aorto-bifemoral: 6.0%, *P* = .026) (aorto-biiliac: 3.0% vs aorto-bifemoral: 6.0%, *P* = .003). Iliac aneurysms were found in 0.5% of patients with aorto-aortic reconstructions, but in 5.9% of patients with biiliac reconstructions (*P* < .001). Patients with bifemoral reconstructions had significantly fewer iliac aneurysms (2.7%) than patients with biiliac reconstructions (*P* = .003) (Table 2).

Limitations of this study include potential coding errors. It is unknown to which level important variables e.g., comorbidities were recorded correctly. It is unclear whether the AOK data represent the standard quality of open aortic aneurysm repair in Germany. The levels of the treating hospitals and the percentage of patients treated in high- and low-volume hospitals were unknown. Information on aneurysm anatomy (juxtarenal/infrarenal) and aneurysm size is not reported in this study.

In conclusion, an aorto-aortic tube grafting should be preferred to aorto-biiliac and aorto-bifemoral reconstructions in OAR whenever morphologically possible. The present study showed that patients with aorto-bifemoral reconstructions had a higher hospital mortality compared to those with other distal anastomoses. Hospital stay was longer, blood transfusion rate and wound infection rate were significantly higher. Long-term outcome was worse. In the multivariate regression analysis, gender was not an independent risk factor for either the perioperative outcome or the long-term outcome.
